# Enhancement of BDNF Concentration and Restoration of the Hypothalamic-Pituitary-Adrenal Axis Accompany Reduced Depressive-Like Behaviour in Stressed Ovariectomised Rats Treated with Either Tualang Honey or Estrogen

**DOI:** 10.1155/2014/310821

**Published:** 2014-01-16

**Authors:** Badriya Al-Rahbi, Rahimah Zakaria, Zahiruddin Othman, Asma' Hassan, Asma Hayati Ahmad

**Affiliations:** ^1^Institute of Health Sciences, Muscat P.O. Box 3720, Ruwi Code 112, Oman; ^2^Department of Physiology, School of Medical Sciences, Universiti Sains Malaysia, 16150 Kubang Kerian, Kelantan, Malaysia; ^3^Department of Psychiatry, School of Medical Sciences, Universiti Sains Malaysia, 16150 Kubang Kerian, Kelantan, Malaysia; ^4^Department of Anatomy, School of Medical Sciences, Universiti Sains Malaysia, 16150 Kubang Kerian, Kelantan, Malaysia

## Abstract

A possible interaction between glucocorticoids and estrogen-induced increases in brain-derived-neurotrophic factor (BDNF) expression in enhancing depressive-like behaviour has been documented. Here we evaluated the effects of Tualang honey, a phytoestrogen, and 17**β**-estradiol (E2) on the depressive-like behaviour, stress hormones, and BDNF concentration in stressed ovariectomised (OVX) rats. The animals were divided into six groups: (i) nonstressed sham-operated control, (ii) stressed sham-operated control, (iii) nonstressed OVX, (iv) stressed OVX, (v) stressed OVX treated with E2 (20 **μ**g daily, sc), and (vi) stressed OVX treated with Tualang honey (0.2 g/kg body weight daily, orally). Two months after surgery, the animals were subjected to social instability stress procedure followed by forced swimming test. Struggling time, immobility time, and swimming time were scored. Serum adrenocorticotropic hormone (ACTH) and corticosterone levels, and the BDNF concentration were determined using commercially available ELISA kits. Stressed OVX rats displayed increased depressive-like behaviour with significantly increased serum ACTH and corticosterone levels, while the BDNF concentration was significantly decreased compared to other experimental groups. These changes were notably reversed by both E2 and Tualang honey. In conclusion, both Tualang honey and E2 mediate antidepressive-like effects in stressed OVX rats, possibly acting via restoration of hypothalamic-pituitary-adrenal axis and enhancement of the BDNF concentration.

## 1. Introduction

Brain-derived-neurotrophic factor (BDNF) is one of several endogenous proteins that play a critical role in the survival, maintenance, and growth of central and peripheral neurons [1]. Importantly, dysfunction of BDNF may play a role in the pathophysiology of various brain diseases such as Huntington's disease [2] and Alzheimer's disease [3]. Reduction in BDNF levels has also been indicated in various mental disorders [4–6] including depression [7, 8]. Decreased expression of BDNF contributes to hippocampal atrophy and neuronal loss in experimental animals [9] and further evidenced by decreased hippocampal volume in depressed patients [10, 11]. Chronic antidepressant treatment induces increases in BDNF level in the hippocampus of rodents [12] and tree shrew [13].

Low ovarian estrogen level increases the susceptibility to depressive disorders in postmenopausal women [4, 6, 14]. Estrogen is widely reported to have antidepressive effects as shown by both human [15, 16] and animal studies [17, 18] and has been suggested as a potential treatment for depressed mood during the postmenopausal period. Several studies demonstrate that estrogen exerts direct modulatory effects on BDNF [19–21]. As upregulation of BDNF is putatively involved in the beneficial effects of several antidepressants, further investigation concerning the detailed mechanisms underlying the hormonal dependent production of BDNF is critical. An earlier study by Sohrabji et al. [22] showed that estrogen regulates the expression of BDNF via the estrogen response element on the BDNF gene. However, the detailed mechanisms concerning steroid hormone-stimulated intracellular signaling and how this regulates BDNF dynamics remain to be elucidated [23].

Persistent increase in glucocorticoids after prolonged exposure to stress may cause extensive damage to the CNS [24], triggering the onset of depression [18]. Since both BDNF and glucocorticoids may be involved in neuronal function as well as the pathophysiology of depression, a possible crosstalk between BDNF and glucocorticoid function has been proposed as one of the mechanisms for depression. Moreover, a large number of preclinical and clinical studies provide evidence supporting the association between stress/depression and hippocampal abnormalities. These include decreased hippocampal neurogenesis as a result of stress conditions [24], its increase after antidepressant treatment [25], and reduction of hippocampal volume in depressed patients [26]. In addition, suppression of hippocampal neurogenesis due to hypothalamic-pituitary-adrenal axis (HPA) hyperactivity is assumed to be one of the major pathways for mood disorders including depression [27].

It is possible that depressive symptoms seen in postmenopausal women with social stress to some extent result from alteration in the BDNF expression and production. However, during the past few years, many women and doctors have revised their opinions of hormone replacement therapy (HRT) for menopausal depressive symptoms [28] and a substantial number of women have discontinued HRT use because of side effects [29]. The main side effects of estrogen-replacement therapy are withdrawal bleeding, bloating, premenstrual irritability, lower abdominal cramps, and breast tenderness [29, 30]. Although many of these troublesome side effects can be managed by adjusting the dose or changing the source of the estrogen or progestin components, postmenopausal women view withdrawal bleeding as the most negative factor [31] influencing their decision to refuse using HRT.

Tualang honey is a multifloral honey produced by the rock bee species (*Apis dorsata*), which builds hives high up in the branches of Tualang tree (*Koompassia excelsa*). In Malaysia, the honey is used traditionally as a health and antiageing supplement [32]. Recently, our research reported that Tualang honey, a phytoestrogen, improved memory performance in postmenopausal women [33] and stressed ovariectomised (OVX) rats [34], comparable to those receiving 17*β*-estradiol (E2) treatment. This study therefore aims to evaluate the possible relationship between the antidepressive-like effects of Tualang honey and E2 and BDNF concentration in stressed OVX rat model.

## 2. Material and Methods

### 2.1. Animal

Adult female Sprague Dawley rats aged 8 weeks old with body weight of 200 ± 20 g were obtained from the Animal Research and Service Centre (ARASC), Health Campus, Universiti Sains Malaysia (USM), Malaysia. All rats were housed in polypropylene cages (40 × 25 × 16 cm), exposed to 12 hr light-dark cycles, maintained at room temperature of 23°C, and fed with pellet diet and water *ad libitum*. Rats' body weights were recorded daily throughout the study. The rats were randomly divided into six groups of 10 rats in each group as follows: nonstressed sham-operated control, stressed sham-operated control, nonstressed OVX, stressed OVX, stressed OVX treated with 17*β*-estradiol, and stressed OVX treated with Tualang honey. The experimental protocol for this study was approved by the Animal Research Ethics Committee of USM (USM/Animal Ethics Approval/2011/(64)(272)), in accordance with the internationally accepted principles for laboratory animal use and care.

### 2.2. Surgical Procedure

The animals were either ovariectomised or sham-operated under anaesthesia (90 mg/kg ketamine and 5 mg/kg xylazine, intraperitoneally). Following anaesthesia, the animal's abdominal fur was shaved and the area carefully cleaned using chlorhexidine scrub and ethanol 70%. Immediately, a small midline incision (2 cm) was made on the dorsal area of lumbar vertebrae 3–5. A high degree of asepsis was maintained throughout the surgery. After surgery, the animals were left under an overhead light source for one hour to avoid hypothermia. Following surgical procedure, the animals were given postoperative care by isolating each animal in a clean cage for 10 days to avoid any infighting that could cause bleeding or poor healing. After 10 days of postoperative care, the rats were housed in groups of three per cage and left for two months for recovery.

### 2.3. Social Instability Stress Procedure

Two months following surgical procedure, rats were exposed to social instability stress procedure consisting of alternate isolation and crowding phases for 15 days based on previously described model [35]. The experiment was started and ended with isolation phase and each phase lasted 24 hours. For crowding phase, eight rats were cohabited in one cage and each group consisted of three males and five females. Male rats were included to mimic normal social behaviour in human. Social contacts between the group members were videotaped for 30 minutes at the beginning of each crowding phase. Biting attacks and dominant postures while fighting for water or food were recorded [36].

### 2.4. Forced Swimming Test

After the social instability stress procedure, each rat was individually tested for depressive-like behaviour using forced swimming test (FST). This test was conducted between 10:00 and 17:00 hours in a brightly lit room. Rats were individually placed in glass cylinders (40 cm in height and 18 cm in diameter) filled with water up to 30 cm in depth. The water level was purposely set higher than that in the procedure described by Porsolt and colleagues [37] in order to prevent the rats from supporting themselves by touching the bottom with their hind limbs or tail during the swimming sessions.

Rats underwent two training sessions lasting 15 minutes per session for two consecutive days. This was followed by a 5-minute test session 24 hours after the second session. Three types of behaviour were scored: (1) immobility: floating in the water, making only movements necessary to keep the head above the water, (2) swimming: making active swimming motions, and (3) struggling: making active attempts to escape from the cylinder, including visual searching for escape routes and diving [37]. After the test, rats were gently dried with a towel and returned to their home cage. Three types of behaviour were scored using a time-sampling technique that has previously been shown to be both valid and reliable in different behavioural studies [36]. Upon completion of FST, rats were sacrificed immediately by rapid decapitation.

After sacrifice, ovariectomy was verified by visual inspection, uterine weights were determined and 10 mL of trunk blood samples was collected immediately. All blood samples were left to clot for 2 hours prior to centrifugation for 15 minutes at 4000 rpm/minutes (EBA 21, Hettich GmbH & Co. KG, Tuttlingen, Germany). Approximately 3 mL of serum was collected and stored at –20°C until assay. Brain from each rat was quickly removed and carefully dissected in ice-cold saline, and brain homogenates were prepared.

### 2.5. Estimation of Serum ACTH and Corticosterone Levels

Serum adrenocorticotropic hormone (ACTH) and corticosterone levels were measured using specific ELISA kit (Creative Diagnostics, Shirley, NY, USA) according to the manufacturer's instructions. Briefly, 100 *μ*L of serum sample was added to each well followed by 100 *μ*L of enzyme-labelled ACTH/corticosterone. The plate was incubated at 37°C for 90 min. Following incubation, the wells were carefully washed. Then, 100 *μ*L of biotin-antibody working solution was added to each well and then incubated at 37°C for 60 min. Following three washes, 100 *μ*L of horseradish peroxidase (HRP) was added to each well and then incubated at 37°C for 30 min. Next, 100 *μ*L of 3,3′,5,5′-tetramethylbenzidine (TMB) reagent was added to each well and incubated at room temperature for 20 min, which resulted in the development of a color change. The colour change was then stopped with the addition of 100 *μ*L of stop solution. The absorbance was measured at 450 nm using a spectrophotometer (Thermo Fisher Scientific Inc., Waltham, MA, USA).

### 2.6. Preparation of Brain Homogenates

The left hemisphere was used to prepare brain homogenates (10% w/v) in ice-cold 0.1 M phosphate-buffered saline (PBS, pH 7.4). The brain homogenates were centrifuged at 10,000 ×g for 10 minutes and stored at −80°C until assay.

### 2.7. Protein Concentration

Following homogenization, an aliquot was removed from each sample to determine its protein concentration using the Bradford method [38]. Briefly, protein concentration was quantified by comparing the colorimetric intensity of the reaction product from each sample with a series of protein standards. All BDNF levels were normalized to their total protein concentration in the sample in order to account for possible differences in protein concentrations between samples. All data were expressed as pg/*μ*g protein.

### 2.8. Measurement of BDNF Concentration

BDNF concentration was measured using commercially available ELISA kits (Cusabio Biotech Co., Ltd., Newark, DE, USA) according to the manufacturer's instructions. Briefly, 100 *μ*L of sample or standard was added to each well and incubated for 90 minutes at 37.5°C. The plate was washed four times with sample diluent. 100 *μ*L of anti-BNDF antibody was added to each well and incubated for 60 minutes at 37.5°C. After a series of washing, 100 *μ*L of HRP-avidin working solution was added to each well and incubated for 30 minutes at 37.5°C. Colorimetric detection of peroxidase activity was achieved by adding 90 *μ*L of tetramethylbenzidine, resulting in blue colour change. The reactions were terminated by adding 50 *μ*L of stop solution to each well. Finally, the amount of BDNF was determined using a spectrophotometer at 450 nm absorbance (Thermo Fisher Scientific Inc., Waltham, MA, USA). The standard curve represents a direct relationship between optical density and BDNF level. BDNF concentration was expressed as pg per mL.

### 2.9. Statistical Analysis

Data were analysed using Statistical Package for the Social Sciences (SPSS) version 20. Two-way analyses of variance (ANOVA) was utilized to examine the effects of stress (stressed versus nonstressed) and surgery (sham-operated versus OVX) conditions on all parameters assessed in this study. One-way ANOVA followed by post-hoc Tukey test was utilized to evaluate the effects of Tualang honey and estradiol on all parameters assessed in this study. Pearson's correlation coefficient was used to test the correlation between body weight and depressive-like behaviour. Data were presented as the mean ± standard error of means (SEM). Probability values of less than 5% (*P* < 0.05) were considered statistically significant.

## 3. Results

### 3.1. Serum ACTH and Corticosterone Levels

The serum ACTH and corticosterone levels were significantly higher in stressed compared to nonstressed groups, that is, 56.952 ± 1.89 versus 45.789 ± 1.200 pg/mL and 5661.905 ± 163.951 versus 4611.000 ± 169.430 pg/mL, respectively.

### 3.2. Depressive-Like Behaviour

Our two-way ANOVA results showed significant main effects of surgery on all depressive-like behaviour such as total swimming time (*F*(1, 36) = 67.49, *P* < 0.001), immobility time (*F*(1, 36) = 140.36, *P* < 0.001), and struggling time (*F*(1, 36) = 146.14, *P* < 0.001). Meanwhile, the main effects of stress were significant on some depressive-like behaviour such as immobility time (*F*(1, 36) = 4.45, *P* < 0.05) and struggling time (*F*(1, 36) = 4.87, *P* < 0.05), but not on swimming time (*F*(1, 36) = 0.26, *P* > 0.05).

In addition, results showed significant surgery and stress interaction on all depressive-like behaviour such as immobility time (*F*(1, 36) = 14.41, *P* < 0.01), struggling time (*F*(1, 36) = 6.13, *P* < 0.05), and swimming time (*F*(1, 36) = 8.83, *P* < 0.01). The results of two-way ANOVA are summarized in Table 1.

**Figure 1:**
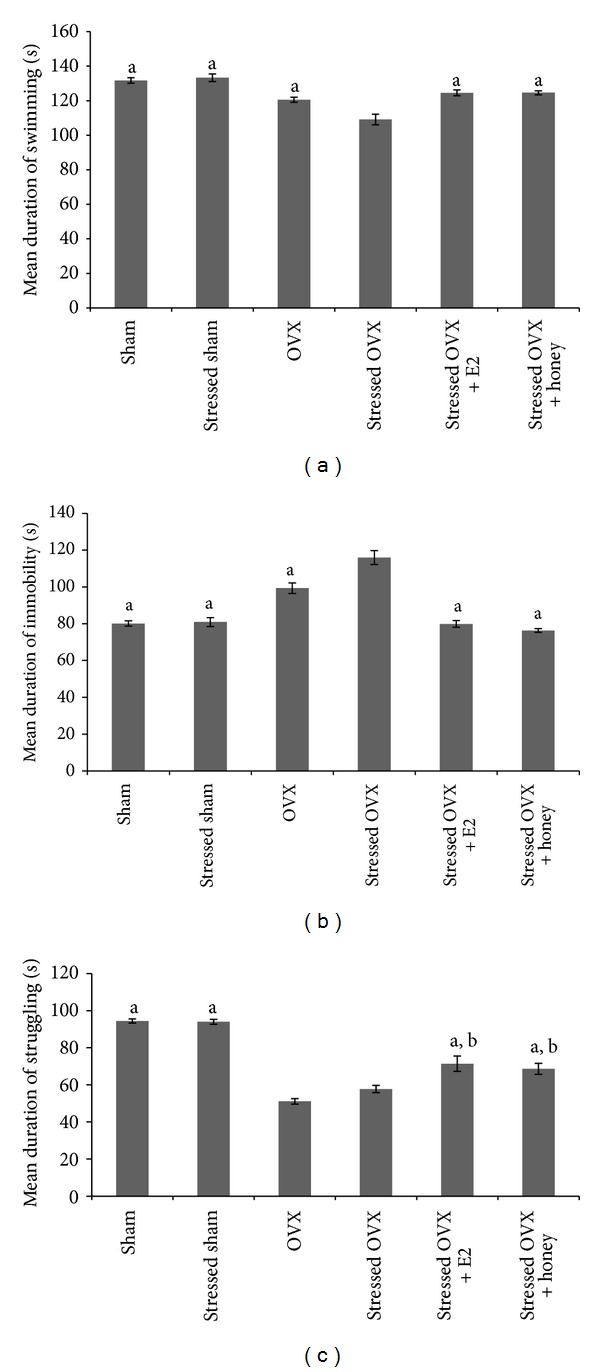
Effects of E2 and Tualang honey treatments on depressive-like behaviour. (a) Swimming time (b) immobility time, and (c) struggling time/5 minutes. Each column represents the mean ± SEM of 10 rats; ^a^ *P* < 0.05 compared with the stressed OVX group; ^b^ *P* < 0.05 compared with the sham group.

The mean immobility time (*F*(5, 56) = 41.79, *P* < 0.001), struggling time (*F*(5, 56) = 55.61, *P* < 0.001), and swimming time (*F*(5, 56) = 19.74, *P* < 0.001) were significantly different among the groups as shown in Figure 1. The post-hoc analysis revealed significant decrease in the mean immobility time and significant increase in the mean swimming time of stressed OVX treated with either E2 or Tualang honey compared to untreated stressed OVX rats. The decrease in the mean immobility time and increase in mean swimming time of both treated groups were comparable to those of sham-operated control rats. For the mean struggling time, there was a significant increase in the stressed OVX treated with either E2 or Tualang honey compared to untreated stressed or nonstressed OVX but the values remained lower than those of sham-operated control rats.

### 3.3. BDNF Concentration

There was a significant main effect of surgery (*F*(1, 36) = 67.49, *P* < 0.001), but not stress (*F*(1, 36) = 0.24, *P* > 0.05) on the BDNF concentration in the brain homogenate. A significant stress and surgery interaction was found on the BDNF concentration in the brain homogenate (*F*(1, 36) = 8.83, *P* < 0.05).

**Figure 2:**
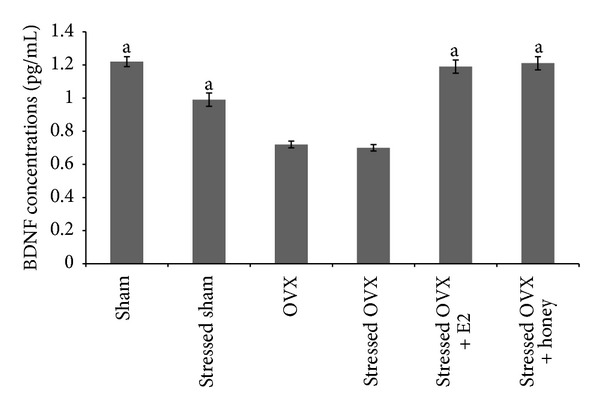
Effects of Tualang honey and E2 treatments on BDNF concentrations. Each column represents the mean ± SEM of 10 rats; ^a^ *P* < 0.05 compared with the stressed OVX group.

One-way ANOVA showed that the mean BDNF concentration in the brain homogenate was significantly different among the groups (*F*(5, 54) = 34.46, *P* < 0.001). Both E2 and Tualang honey increased the mean BDNF concentration in the brain homogenate of stressed OVX comparable to that of sham-operated control rats as shown in Figure 2.

### 3.4. Correlation between Body Weight and Immobility Time, Struggling Time, and Swimming Time

The body weight gain was significantly higher in OVX rats compared to sham-operated controls (59.60 ± 2.72 versus 24.20 ± 0.25 g). There was no significant correlation between body weight gain and depressive-like behaviour as shown in Table 2.

## 4. Discussion

The possibility of ovariectomy-induced weight gain [39] associated with longer immobility time and shorter struggling time and swimming time was excluded by evaluating the correlation between body weight and depressive-like behaviour in the OVX rats using the Pearson product-moment correlation. We found no significant correlation between these variables. Although body weight in the ovariectomised group was significantly greater than that in the sham-operated group, this weight gain did not participate in the prolongation of immobility or reduction in swimming time and struggling time induced by ovariectomy. It was earlier noted that the duration of immobility was increased while swimming and struggling activities were decreased in ovariectomised compared to non-ovariectomised rats [40].

We found that stressed ovariectomised rats displayed more depressive-like behaviour compared to the other experimental groups consistent with findings from previous studies [41–43]. Our results also revealed significant interaction between stress and OVX on all parameters of depressive-like behaviour supporting previous findings regarding interactions between gonadal and adrenal hormones in hippocampal function [44–52]. It was previously suggested that deprivation of ovarian hormones for a long period of time magnifies the effect of chronic unpredictable stress on depressive-like behaviour in mice [43].

More interestingly, our data also showed that Tualang honey or E2-treated stressed OVX rats had significant reduction in ACTH and corticosterone levels, as well as depressive-like behaviour compared to those of untreated stressed OVX rats. Estrogen has been shown to produce an antidepressive-like effect during the forced swimming test in female rats [53–57]. Furthermore, the link between estrogen and BDNF expression and production has been proposed. Ovariectomy decreases levels of BDNF mRNA, and estradiol treatment reverses the effect in female rat hippocampus [19, 22, 58]. It was subsequently demonstrated that administration of estradiol to gonadectomized male rat pups restores BDNF expression in hippocampal pyramidal cells; an estrogen response element (ERE) mechanism is implicated because estrogen receptor-*α* (ER*α*) colocalizes with BDNF [59]. Taken together, findings from these studies suggest that the actions of estradiol in the hippocampus include regulation of BDNF. Our findings of increased BDNF concentration and reduced depressive-like behaviour following E2 treatment in stressed OVX rats are further affirmation of this.

So far there is no report of the effects of honey on depressive-like behaviour or on the BDNF concentration. In the present study, apart from reducing depressive-like behaviour, Tualang honey-treated stressed OVX rats also increased the BDNF concentration comparable to those of E2-treated stressed OVX rats. Since Tualang honey is a phytoestrogen rich in flavonoids, it is possible that the mechanisms of antidepressive-like actions are similar to other phytochemical food rich in flavonoids such as green tea, blueberry, and Ginkgo Biloba which have been shown to increase hippocampal BDNF levels [60–64]. Recently, another study reported that flavonoids-induced synthesis and secretion of BDNF in cultured rat astrocytes are mediated by the estrogen receptor [65], providing further indication of the possible underlying mechanism.

Stress-induced reductions of BDNF may produce a risk for depression, while restoration of HPA axis and BDNF homeostasis following E2 and Tualang honey treatment may be responsible for the improvement in mood [66–71]. The ability of BDNF to reverse depressive-like behaviour in the animal models may be attributed to increased monoaminergic activity within the central nervous system that in turn compensates for the decreased levels of BDNF [72–74].

In conclusion, both stress hormones and estrogen deficiency reduced the BDNF concentration and increased depressive-like behaviour. The present study revealed that treatment with either E2 or Tualang honey was able to reverse these effects. It is possible that the antidepressive-like effects of Tualang honey and E2 seen in our stressed OVX rats model act via restoration of HPA axis and enhancement of BDNF concentration.

## Figures and Tables

**Table 1:** Effects of surgery and stress on depressive-like behaviour.

Group	Duration (seconds)		
	Swimming	Immobility	Struggling
Sham-operated			
Nonstressed	131.70 ± 1.59	78.00 ± 2.0	94.41 ± 1.08
Stressed	133.30 ± 2.19	72.10 ± 2.50	94.12 ± 1.33
OVX			
Nonstressed	120.28 ± 1.41	115.30 ± 1.54	51.10 ± 1.49
Stressed	109.70 ± 3.40*	18.91 ± 2.66	57.70 ± 3.28*
Stress effect	*P* > 0.05	*P* < 0.05	*P* < 0.05
Surgery effect	*P* < 0.001	*P* < 0.001	*P* < 0.001
Stress × surgery interaction	*P* < 0.05	*P* < 0.05	*P* < 0.05

Each value represents the mean ± SEM of 10 rats, significantly different from nonstressed group at *P < 0.05.

**Table 2:** Correlation between the body weight gain and depressive-like behaviour.

Depressive-like behaviour (seconds)	Body weight gain (gram)
Swimming	−0.266
Struggling	−0.253
Immobility	0.172

Body weight gain is the difference in body weight after surgical procedure and at sacrifice. Each value represents the Pearson correlation coefficients (*r*). *Significant difference at *P* < 0.05.

## References

[1] Garcia LSB, Comim CM, Valvassori SS (2008). Acute administration of ketamine induces antidepressant-like effects in the forced swimming test and increases BDNF levels in the rat hippocampus. *Progress in Neuro-Psychopharmacology and Biological Psychiatry*.

[2] Canals JM, Pineda JR, Torres-Peraza JF (2004). Brain-derived neurotrophic factor regulates the onset and severity of motor dysfunction associated with enkephalinergic neuronal degeneration in Huntington’s disease. *Journal of Neuroscience*.

[3] Zhang H, Ozbay F, Lappalainen J (2006). Brain Derived Neurotrophic Factor (BDNF) gene variants and Alzheimer’s disease, affective disorders, posttraumatic stress disorder, schizophrenia, and substance dependence. *American Journal of Medical Genetics B*.

[4] Coope J (1981). Is oestrogen therapy effective in the treatment of menopausal depression?. *Journal of the Royal College of General Practitioners*.

[5] Seeman MV (1997). Psychopathology in women and men: focus on female hormones. *The American Journal of Psychiatry*.

[6] Hayward C, Sanborn K (2002). Puberty and the emergence of gender differences in psychopathology. *Journal of Adolescent Health*.

[7] Shimizu E, Hashimoto K, Okamura N (2003). Alterations of serum levels of Brain-Derived Neurotrophic Factor (BDNF) in depressed patients with or without antidepressants. *Biological Psychiatry*.

[8] Franklin TB, Perrot-Sinal TS (2006). Sex and ovarian steroids modulate Brain-Derived Neurotrophic Factor (BDNF) protein levels in rat hippocampus under stressful and non-stressful conditions. *Psychoneuroendocrinology*.

[9] McEwen BS (1999). Stress and hippocampal plasticity. *Annual Review of Neuroscience*.

[10] Duman RS, Malberg J, Nakagawa S (2001). Regulation of adult neurogenesis by psychotropic drugs and stress. *Journal of Pharmacology and Experimental Therapeutics*.

[11] Manji HK, Drevets WC, Charney DS (2001). The cellular neurobiology of depression. *Nature Medicine*.

[12] Nibuya M, Nestler EJ, Duman RS (1996). Chronic antidepressant administration increases the expression of cAMP response element binding protein (CREB) in rat hippocampus. *Journal of Neuroscience*.

[13] Czéh B, Michaelis T, Watanabe T (2001). Stress-induced changes in cerebral metabolites, hippocampal volume, and cell proliferation are prevented by antidepressant treatment with tianeptine. *Proceedings of the National Academy of Sciences of the United States of America*.

[14] Young EA, Altemus M (2004). Puberty, ovarian steroids, and stress. *Annals of the New York Academy of Sciences*.

[15] Schmidt PJ, Nieman L, Danaceau MA (2000). Estrogen replacement in perimenopause-related depression: a preliminary report. *American Journal of Obstetrics and Gynecology*.

[16] Cohen LS, Soares CN, Poitras JR, Prouty J, Alexander AB, Shifren JL (2003). Short-term use of estradiol for depression in perimenopausal and postmenopausal women: a preliminary report. *The American Journal of Psychiatry*.

[17] Walf AA, Rhodes ME, Frye CA (2004). Antidepressant effects of ER*β*-selective estrogen receptor modulators in the forced swim test. *Pharmacology Biochemistry and Behavior*.

[18] Rocha BA, Fleischer R, Schaeffer JM, Rohrer SP, Hickey GJ (2005). 17*β*-Estradiol-induced antidepressant-like effect in the Forced Swim Test is absent in estrogen receptor-*β* knockout (BERKO) mice. *Psychopharmacology*.

[19] Singh M, Meyer EM, Simpkins JW (1995). The effect of ovariectomy and estradiol replacement on brain-derived neurotrophic factor messenger ribonucleic acid expression in cortical and hippocampal brain regions of female Sprague-Dawley rats. *Endocrinology*.

[20] Gibbs RB (1999). Treatment with estrogen and progesterone affects relative levels of brain-derived neurotrophic factor mRNA and protein in different regions of the adult rat brain. *Brain Research*.

[21] Jezierski MK, Sohrabji F (2000). Region- and peptide-specific regulation of the neurotrophins by estrogen. *Molecular Brain Research*.

[22] Sohrabji F, Miranda RCG, Toran-Allerand CD (1995). Identification of a putative estrogen response element in the gene encoding brain-derived neurotrophic factor. *Proceedings of the National Academy of Sciences of the United States of America*.

[23] Numakawa T, Yokomaku D, Richards M, Hori H, Adachi N, Kunugi H (2010). Functional interactions between steroid hormones and neurotrophin BDNF. *World Journal of Biological Chemistry*.

[24] McEwen BS, Sapolsky RM (1995). Stress and cognitive function. *Current Opinion in Neurobiology*.

[25] Armario A, Gavalda A, Marti O (1988). Forced swimming test in rats: effects of desipramine administration and the period of exposure to the test on struggling behavior, swimming, immobility and defecation rate. *European Journal of Pharmacology*.

[26] Grønli J, Bramham C, Murison R (2006). Chronic mild stress inhibits BDNF protein expression and CREB activation in the dentate gyrus but not in the hippocampus proper. *Pharmacology Biochemistry and Behavior*.

[27] Murakami S, Imbe H, Morikawa Y, Kubo C, Senba E (2005). Chronic stress, as well as acute stress, reduces BDNF mRNA expression in the rat hippocampus but less robustly. *Neuroscience Research*.

[28] Hickey M, Davis SR, Sturdee DW (2005). Treatment of menopausal symptoms: what shall we do now?. *The Lancet*.

[29] Archer DF (2001). The effect of the duration of progestin use on the occurrence of endometrial cancer in postmenopausal women. *Menopause*.

[30] Rasgon NL, Altshuler LL, Fairbanks LA (2002). Estrogen replacement therapy in the treatment of major depressive disorder in perimenopausal women. *Journal of Clinical Psychiatry*.

[31] Judd HL, Cleary RE, Creasman WT (1981). Estrogen replacement therapy. *Obstetrics and Gynecology*.

[32] Ahmed S, Othman NH (2013). Review of the medicinal effects of Tualang Honey and a comparison with Manuka Honey. *Malaysian Journal of Medical Sciences*.

[33] Othman Z, Shafin N, Zakaria R, Hussain NHN, Mohammad WMZW (2011). Improvement in immediate memory after 16 weeks of tualang honey (Agro Mas) supplement in healthy postmenopausal women. *Menopause*.

[34] Al-Rahbi B, Zakaria R, Othman Z, Hassan A, Mohd Ismail ZI, Muthuraju S (2013). Tualang honey supplement improves memory performance and hippocampal morphology in stressed overiectomized rats. *Acta Histochemica*.

[35] Haller J, Fuchs E, Halász J, Makara GB (1999). Defeat is a major stressor in males while social instability is stressful mainly in females: towards the development of a social stress model in female rats. *Brain Research Bulletin*.

[36] Detke MJ, Rickels M, Lucki I (1995). Active behaviors in the rat forced swimming test differentially produced by serotonergic and noradrenergic antidepressants. *Psychopharmacology*.

[37] Porsolt RD, Anton G, Blavet N, Jalfre M (1978). Behavioural despair in rats: a new model sensitive to antidepressant treatments. *European Journal of Pharmacology*.

[38] Bradford MM (1976). A rapid and sensitive method for the quantitation of microgram quantities of protein utilizing the principle of protein dye binding. *Analytical Biochemistry*.

[39] Ainslie DA, Morris MJ, Wittert G, Turnbull H, Proietto J, Thorburn AW (2001). Estrogen deficiency causes central leptin insensitivity and increased hypothalamic neuropeptide Y. *International Journal of Obesity*.

[40] Al-Rahbi B, Zakaria R, Othman Z, Hassan A, Muthuraju S, Mohammad WMZW (2013). Mood and memory function in ovariectomised rats exposed to social instability stress. *BioMed Research International*.

[41] Okada M, Hayashi N, Kometani M, Nakao K, Inukai T (1997). Influences of ovariectomy and continuous replacement of 17*β*-estradiol on the tail skin temperature and behavior in the forced swimming test in rats. *Japanese Journal of Pharmacology*.

[42] Bekku N, Yoshimura H (2005). Animal model of menopausal depressive-like state in female mice: prolongation of immobility time in the forced swimming test following ovariectomy. *Psychopharmacology*.

[43] Lagunas N, Calmarza-Font I, Diz-Chaves Y, Garcia-Segura LM (2010). Long-term ovariectomy enhances anxiety and depressive-like behaviors in mice submitted to chronic unpredictable stress. *Hormones and Behavior*.

[44] Galea LAM, McEwen BS, Tanapat P, Deak T, Spencer RL, Dhabhar FS (1997). Sex differences in dendritic atrophy of CA3 pyramidal neurons in response to chronic restraint stress. *Neuroscience*.

[45] Wood GE, Shors TJ (1998). Stress facilitates classical conditioning in males, but impairs classical conditioning in females through activational effects of ovarian hormones. *Proceedings of the National Academy of Sciences of the United States of America*.

[46] Bowman RE, Zrull MC, Luine VN (2001). Chronic restraint stress enhances radial arm maze performance in female rats. *Brain Research*.

[47] Shors TJ, Chua C, Falduto J (2001). Sex differences and opposite effects of stress on dendritic spine density in the male versus female hippocampus. *Journal of Neuroscience*.

[48] Wood GE, Beylin AV, Shors TJ (2001). The contribution of adrenal and reproductive hormones to the opposing effects of stress on trace conditioning in males versus females. *Behavioral Neuroscience*.

[49] Shors TJ, Falduto J, Leuner B (2004). The opposite effects of stress on dendritic spines in male vs. female rats are NMDA receptor-dependent. *European Journal of Neuroscience*.

[50] McLaughlin KJ, Baran SE, Wright RL, Conrad CD (2005). Chronic stress enhances spatial memory in ovariectomized female rats despite CA3 dendritic retraction: possible involvement of CA1 neurons. *Neuroscience*.

[51] Shors TJ, Mathew J, Sisti HM, Edgecomb C, Beckoff S, Dalla C (2007). Neurogenesis and helplessness are mediated by controllability in males but not in females. *Biological Psychiatry*.

[52] McLaughlin KJ, Wilson JO, Harman J (2010). Chronic 17*β*-estradiol or cholesterol prevents stress-induced hippocampal CA3 dendritic retraction in ovariectomized female rats: possible correspondence between CA1 spine properties and spatial acquisition. *Hippocampus*.

[53] Bernardi M, Vergoni AV, Sandrini M, Tagliavini S, Bertolini A (1989). Influence of ovariectomy, estradiol and progesterone on the behavior of mice in an experimental model of depression. *Physiology and Behavior*.

[54] Rachman IM, Unnerstall JR, Pfaff DW, Cohen RS (1998). Estrogen alters behavior and forebrain c-fos expression in ovariectomized rats subjected to the forced swim test. *Proceedings of the National Academy of Sciences of the United States of America*.

[55] Galea LAM, Wide JK, Barr AM (2001). Estradiol alleviates depressive-like symptoms in a novel animal model of post-partum depression. *Behavioural Brain Research*.

[56] Frye CA, Walf AA (2002). Changes in progesterone metabolites in the hippocampus can modulate open field and forced swim test behavior of proestrous rats. *Hormones and Behavior*.

[57] Estrada-Camarena E, Fernández-Guasti A, López-Rubalcava C (2003). Antidepressant-like effect of different estrogenic compounds in the forced swimming test. *Neuropsychopharmacology*.

[58] Berchtold NC, Kesslak JP, Pike CJ, Adlard PA, Cotman CW (2001). Estrogen and exercise interact to regulate brain-derived neurotrophic factor mRNA and protein expression in the hippocampus. *European Journal of Neuroscience*.

[59] Solum DT, Handa RJ (2002). Estrogen regulates the development of brain-derived neurotrophic factor mRNA and protein in the rat hippocampus. *Journal of Neuroscience*.

[60] Williams CM, El Mohsen MA, Vauzour D (2008). Blueberry-induced changes in spatial working memory correlate with changes in hippocampal CREB phosphorylation and Brain-Derived Neurotrophic Factor (BDNF) levels. *Free Radical Biology and Medicine*.

[61] Li Q, Zhao HF, Zhang ZF (2009). Long-term administration of green tea catechins prevents age-related spatial learning and memory decline in C57BL/6 J mice by regulating hippocampal cyclic amp-response element binding protein signaling cascade. *Neuroscience*.

[62] Li Q, Zhao HF, Zhang ZF (2009). Long-term green tea catechin administration prevents spatial learning and memory impairment in senescence-accelerated mouse prone-8 mice by decreasing A*β*1-42 oligomers and upregulating synaptic plasticity-related proteins in the hippocampus. *Neuroscience*.

[63] Hou Y, Aboukhatwa MA, Lei D-L, Manaye K, Khan I, Luo Y (2010). Anti-depressant natural flavonols modulate BDNF and beta amyloid in neurons and hippocampus of double TgAD mice. *Neuropharmacology*.

[64] Rendeiro C, Vauzour D, Rattray M (2013). Dietary levels of pure flavonoids improve spatial memory performance and increase hippocampal brain-derived neurotrophic factor. *PLoS ONE*.

[65] Xu SL, Bi CWC, Choi RCY (2013). Flavonoids induce the synthesis and secretion of neurotrophic factors in cultured rat astrocytes: a signaling response mediated by estrogen receptor. *Evidence-Based Complementary and Alternative Medicine*.

[66] Russo-Neustadt A, Beard RC, Cotman CW (1999). Exercise, antidepressant medications, and enhanced brain derived neurotrophic factor expression. *Neuropsychopharmacology*.

[67] Altar CA, Whitehead RE, Chen R, Wörtwein G, Madsen TM (2003). Effects of electroconvulsive seizures and antidepressant drugs on brain-derived neurotrophic factor protein in rat brain. *Biological Psychiatry*.

[68] Garza AA, Ha TG, Garcia C, Chen MJ, Russo-Neustadt AA (2004). Exercise, antidepressant treatment, and BDNF mRNA expression in the aging brain. *Pharmacology Biochemistry and Behavior*.

[69] Lee B-H, Kim H, Park S-H, Kim Y-K (2007). Decreased plasma BDNF level in depressive patients. *Journal of Affective Disorders*.

[70] MacQueen G, Frodl T (2011). The hippocampus in major depression: evidence for the convergence of the bench and bedside in psychiatric research. *Molecular Psychiatry*.

[71] Molendijk ML, Bus BAA, Spinhoven P (2011). Serum levels of brain-derived neurotrophic factor in major depressive disorder: state-trait issues, clinical features and pharmacological treatment. *Molecular Psychiatry*.

[72] Morinobu S, Duman RS (1993). Induction of BDNF mRNA by electroconvulsive seizure (ECS) and antidepressants in rat frontal. *Society for Neuroscience*.

[73] Nibuya M, Rydelek-Fitzgerald L, Morinobu S, Russel DS, Nestler EJ, Duman RS (1994). Induction of BDNF and trkB by electroconvulsive seizure (ECS): regional regulation and role of CREB. *Society for Neuroscience*.

[74] Siuciak JA, Lewis DR, Wiegand SJ, Lindsay RM (1996). Antidepressant-like effect of Brain-Derived Neurotrophic Factor (BDNF). *Pharmacology Biochemistry and Behavior*.

